# Supplementary material for: Imprinted Cdkn1c genomic locus cell-autonomously promotes cell survival in cerebral cortex development
Source: Nat Commun. 2020 Jan 10;11:195. doi: 10.1038/s41467-019-14077-2 (PMC6954230; doi:10.1038/s41467-019-14077-2)
Supplement: Supplementary file 1 — Supplementary Information [file 41467_2019_14077_MOESM1_ESM.pdf]

Supplementary Information

Imprinted *Cdkn1c* Genomic Locus Cell-Autonomously Promotes Cell Survival in Cerebral Cortex Development

Laukoter et al.

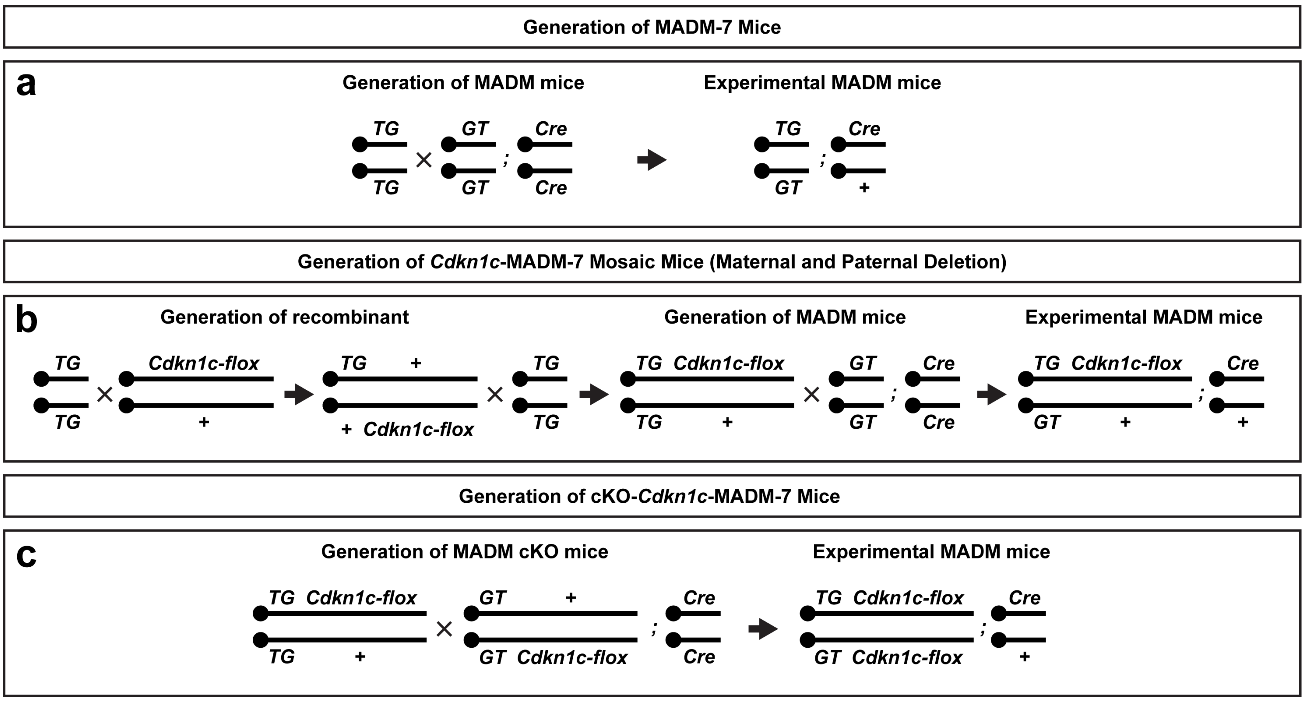

Supplementary Figure 1. Breeding scheme for the generation of mosaic- and cKO-MADM mice with Chr. 7 UPD. (a-c) Breeding scheme for the generation of experimental MADM-7 **(a)**, *Cdkn1c*-MADM-7 (maternal and paternal deletion) **(b)**, and cKO-*Cdkn1c*-MADM-7 **(c)** animals.

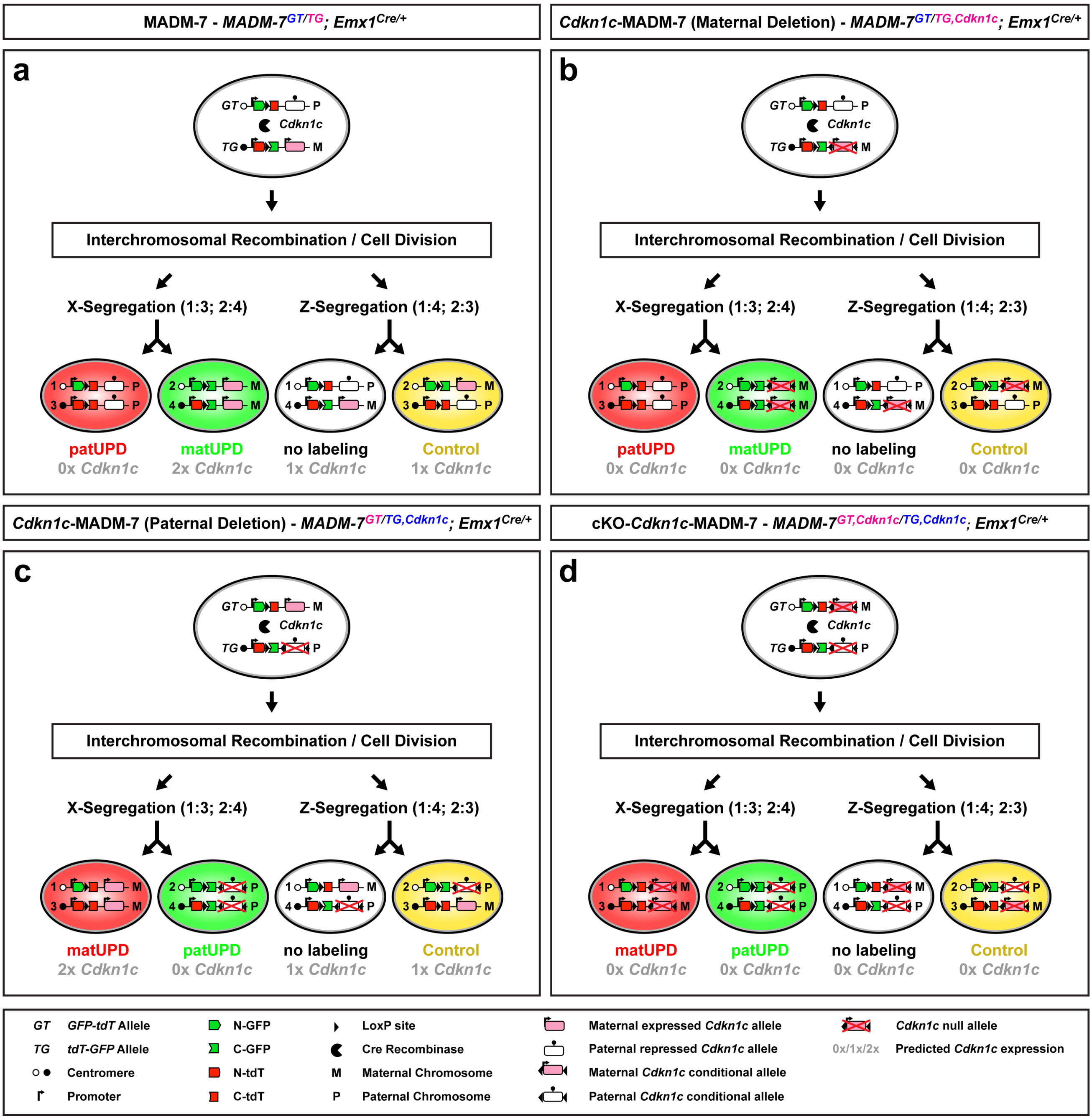

Supplementary Figure 2. Experimental paradigm and MADM scheme for analyzing imprinted *Cdkn1c*. **(a)**, MADM-7 scheme for imprinted *Cdkn1c*. Imprinting (silencing) of the paternal *Cdkn1c* allele (white) and expression from the maternal allele (pink) are indicated in the cell before MADM is induced. The GT- or TG-MADM cassette is located on the paternal or maternal chromosome, respectively. Different MADM events would produce labeled cells with the expressed *Cdkn1c* gene dose shown at the bottom: 2x *Cdkn1c* in green cells, 1x *Cdkn1c* in yellow and unlabeled cells, and no *Cdkn1c* expression in red cells. **(b)** *Cdkn1c*-MADM-7 scheme for maternal transmission of the floxed/deleted *Cdkn1c* allele. Because the paternal allele is silenced, no *Cdkn1c* expression is predicted in green, red, and yellow cells. **(c)** *Cdkn1c*-MADM-7 scheme for paternal transmission of the floxed/deleted *Cdkn1c* allele. Because the paternal allele is silenced, the *Cdkn1c* expression pattern is predicted to resemble that of MADM-7 (**a**). **(d)** cKO-*Cdkn1c*-MADM-7 scheme for transmission of the floxed/deleted *Cdkn1c* allele from both parents. No *Cdkn1c* expression occurs in green, red, yellow and unlabeled cells of the $Emx1^{+}$ lineage.

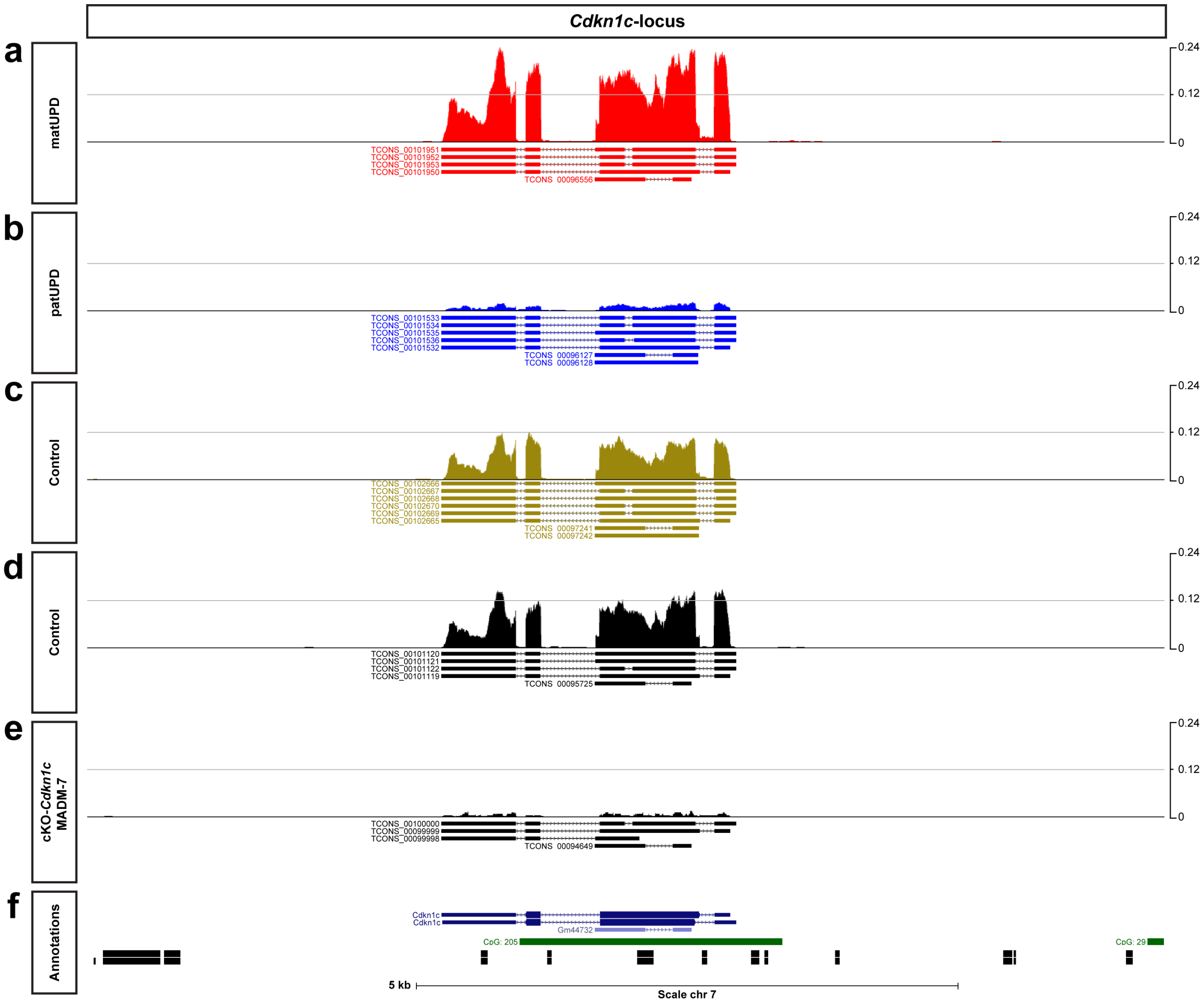

Supplementary Figure 3. Coverage plots of *Cdkn1c* locus in UPD and *Cdkn1c* ablation paradigm. UCSC genome browser (https://genome.ucsc.edu/) display of RNA-seq read coverage as average size normalized coverage per base pair. **(a-f)** *Cdkn1c* locus position Chr7:143455000-143465000 (mm10/GRCm38). **(a-c)** FACS purified E16.5 MADM-7 ($MADM\text{-}7^{GT/TG}$;$Emx1^{Cre/+}$) matUPD cells (a, 7 biological replicates), patUPD cells (b, 7 biological replicates) and matching control cells (c, 8 biological replicates). **(d)** E16.5 Control (7 biological replicates) and **(e)** cKO-*Cdkn1c*-MADM-7 ($MADM\text{-}7^{GT,Cdkn1c/TG,Cdkn1c}$;$Emx1^{Cre/+}$) cells (3 biological replicates). **(f)** UCSC genome browser annotations: Gencode VM23 (top, blue), CpG island (middle, green), RepeatMasker (bottom, black). Note that reference annotation based transcript assembly (TCONS, below coverage plots) did not show any sign of novel transcripts in this region.

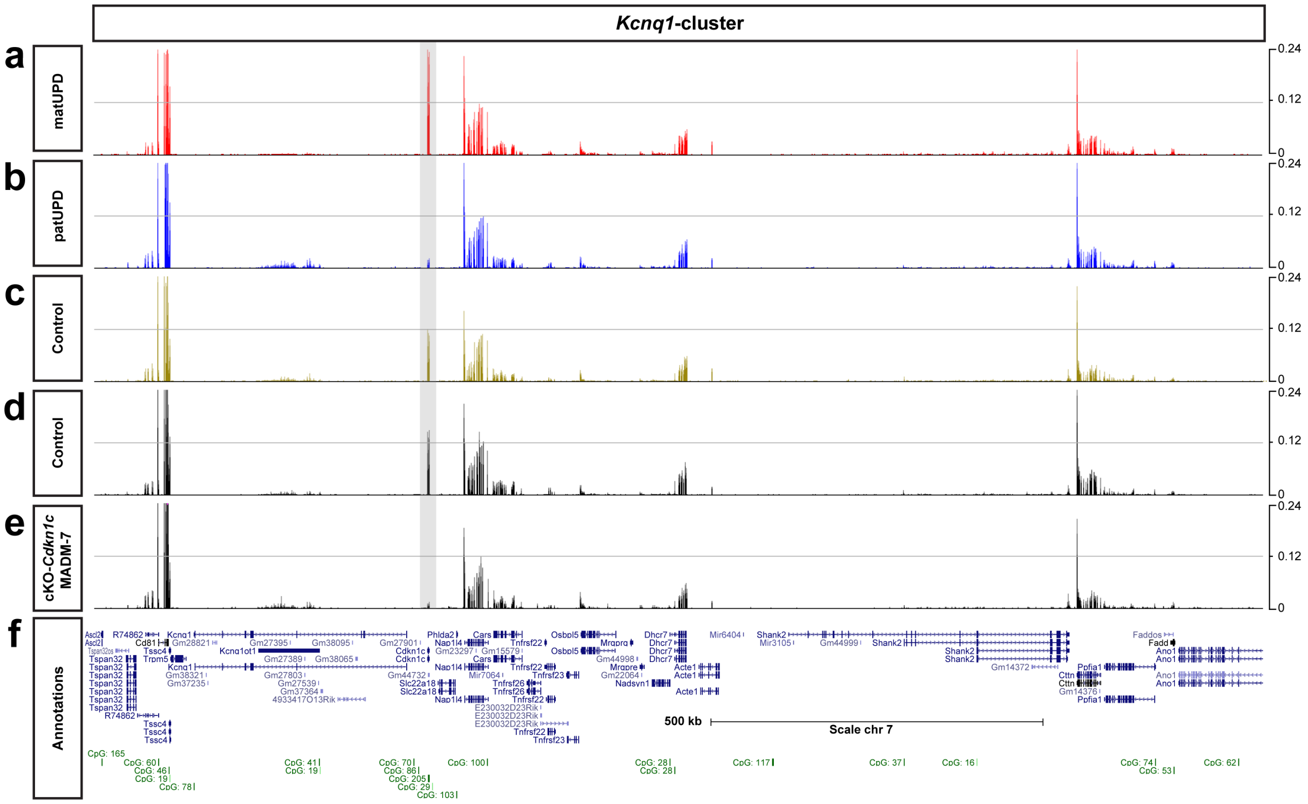

Supplementary Figure 4. Coverage plots of *Kcnq1* cluster in UPD and *Cdkn1c* ablation paradigm. UCSC genome browser (https://genome.ucsc.edu/) display of RNA-seq read coverage as average size normalized coverage per base pair. **(a-f)** *Kcnq1* cluster position Chr7:142966829-144738543 (mm10/GRCm38). **(a-c)** FACS purified E16.5 MADM-7 (*MADM-7*$^{GT/TG}$;*Emx1*$^{Cre/+}$) matUPD cells (a, 7 biological replicates), patUPD cells (b, 7 biological replicates) and matching control cells (c, 8 biological replicates). **(d)** E16.5 Control (7 biological replicates) and **(e)** cKO-*Cdkn1c*-MADM-7 (*MADM-7*$^{GT,Cdkn1c/TG,Cdkn1c}$;*Emx1*$^{Cre/+}$) cells (3 biological replicates). **(f)** UCSC genome browser annotations: Gencode VM23 (top, blue), CpG island (middle, green), RepeatMasker (bottom, black). Note that except for *Cdkn1c* locus (grey box) there are no major differences in read coverage.

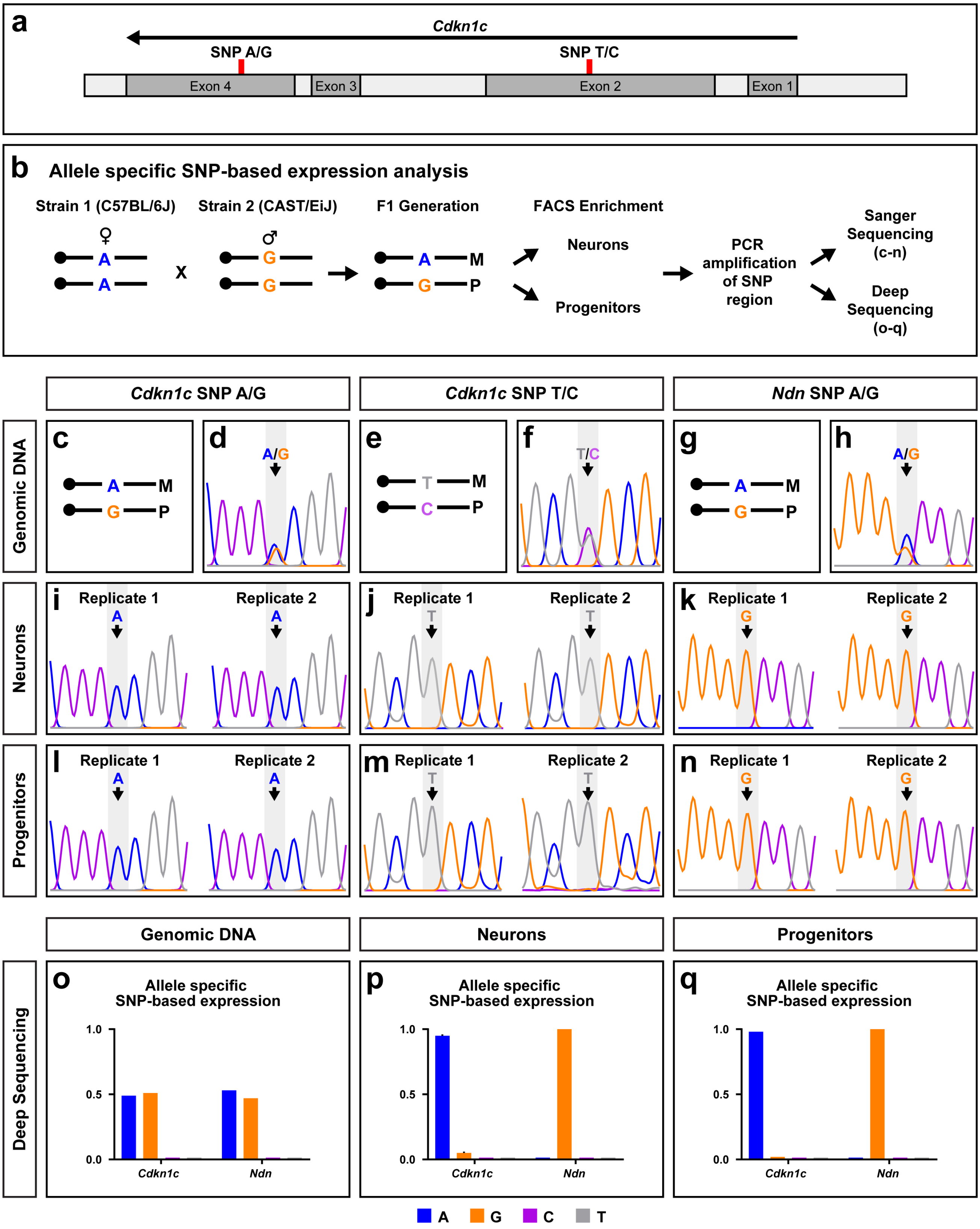

Supplementary Figure 5. Imprinting status of *Cdkn1c* in cortical RGPs and nascent projection neurons. **(a)** Overview of genomic *Cdkn1c* locus. Two SNPs, one in Exon 2 and one in Exon 4 are indicated. **(b)** Strategy for cell-type specific (RGPs and projection neurons) allelic expression analysis using the indicated SNP in Exon 4. Parental strains 1 and 2 [C57BL/6J (B6) and CAST/EiJ (CAST)] strains are crossed for generating F1 B6xCAST hybrids. Genomic DNA is isolated from body tissue and cell suspensions from

cortex of B6xCAST hybrids are sorted by FACS. SNP region in *Cdkn1c* and *Ndn*, respectively was amplified from genomic DNA and cDNA (neurons and RGPs), and subjected to Sanger and deep sequencing. Parental origin of chromosome is indicated (M, maternal; P, paternal). (**c, e, g**) Expected SNP in F1 generation: A/G in *Cdkn1c* exon 4 **(c)**, T/C in *Cdkn1c* exon 2 **(e)** and A/G in *Ndn* **(g)**. **(d, f, h)** Confirmation of SNP presence in genomic DNA by Sanger sequencing: A/G in *Cdkn1c* exon 4 (**d**), T/C in *Cdkn1c* exon 2 (**f**) and A/G in *Ndn* (**h**). Note the presence of both SNP peaks overlapping in the panels with genomic DNA (**d, f, h**). **(i-n)** Qualitative assessment of SNP presence by Sanger sequencing in neurons (**i-k**) and progenitors (**l-n**). Only the maternal SNP (A or T) for *Cdkn1c* is present in all neuron **(i, j)** and RGP replicates **(l, m)** and only the paternal SNP (G) is present for *Ndn* in neurons (**k**) and progenitors (**n**). **(o-q)** Quantitative analysis of SNP presence by deep sequencing in genomic DNA (**o**); and *Cdkn1c* and *Ndn* allelic expression in nascent neurons (**p**) and RGPs (**q**). Note the almost exclusive maternal (*Cdkn1c*) and paternal (*Ndn*) expression in both, progenitors and neurons. Bars and error bars represent mean ± max/min of biological replicates.

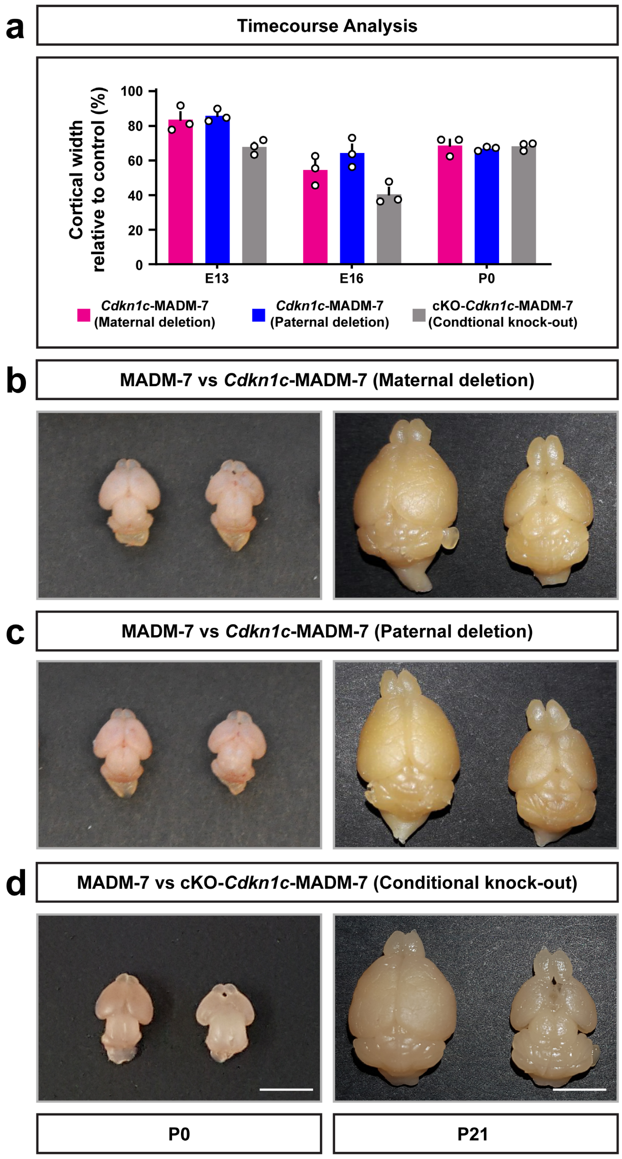

Supplementary Figure 6. Time course analysis of emerging microcephaly upon *Cdkn1c*-deletion. (a) Quantification of cortical width (% relative to MADM-7 serving as control) at E13, E16, and P0 in *Cdkn1c*-MADM-7 with maternal deletion (pink bars), paternal deletion (blue bars) and in cKO-*Cdkn1c*-MADM-7 (grey bars). Data points indicate individual animals (n=3) and values (bars) represent mean ±SEM. **(b)** Photographs of whole brains from *Cdkn1c*-MADM-7 with maternal deletion (right brain in both of the images) with corresponding MADM-7 controls (left brain in both of the images) at P0 (left image) and P21 (right image). **(c)** Photographs of whole brains from *Cdkn1c*-MADM-7 with paternal deletion (right brain in both of the images) with corresponding MADM-7 controls (left brain in both of the images) at P0 (left image) and P21 (right image). **(d)** Photographs of whole brains from cKO-*Cdkn1c*-MADM-7 (right brain in both of the images) with corresponding MADM-7 controls (left brain in both of the images) at P0 (left image) and P21 (right image). Scale bar indicates 1 cm.

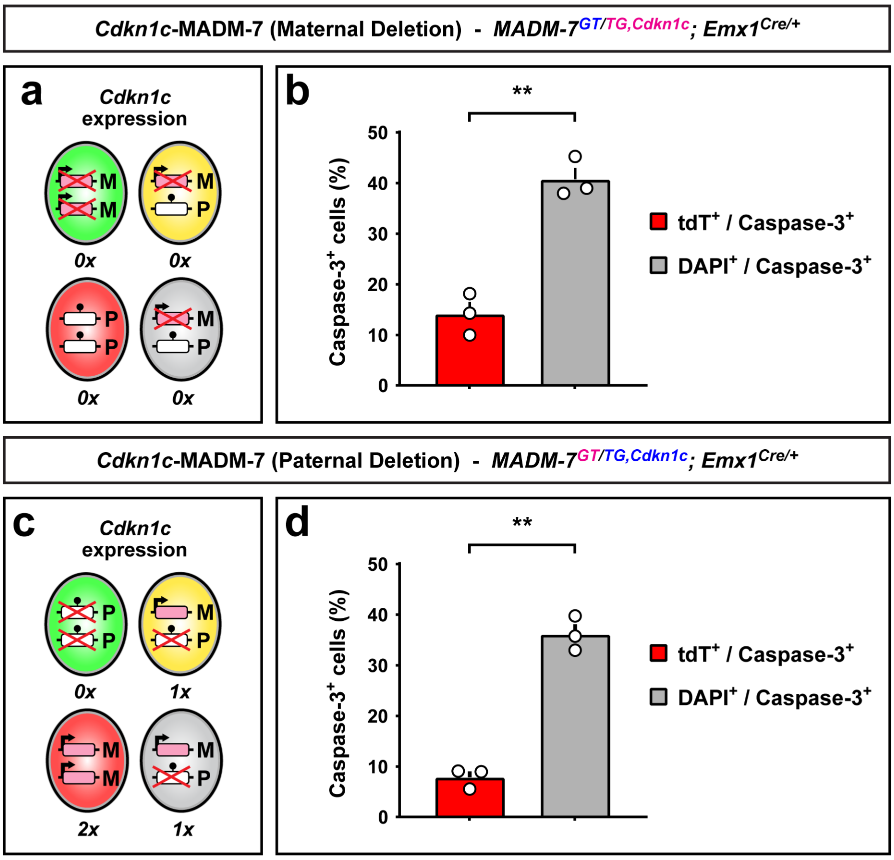

Supplementary Figure 7. Haploinsufficiency of *Cdkn1c* genomic locus. Analysis of *Cdkn1c*-MADM-7 (*MADM-7*$^{GT/TG,Cdkn1c}$;*Emx1*$^{Cre/+}$) with maternal deletion **(a, b)** and *Cdkn1c*-MADM-7 (*MADM-7*$^{GT/TG,Cdkn1c}$;*Emx1*$^{Cre/+}$) with paternal deletion **(c, d)** at E13. The parent from which the MADM cassette, and with recombined *Cdkn1c-flox* allele (*Cdkn1c*) was inherited, is indicated in the respective genotypes in pink (mother) and blue (father). Schematics **(a, c)** depict green (GFP^{+}), red (tdT^{+}), yellow (GFP^{+}/tdT^{+}) and unlabeled MADM cells with UPD (red, green) and control cells (yellow, unlabeled). Imprinting (arrow, expression; ball on stick, repression) and expression (0x, 1x, 2x) status is indicated. Conditional deletion of *Cdkn1c* is marked with red cross. Parental origin of chromosome is indicated (M, maternal; P, paternal). Quantification (%) of tdT^{+}/Caspase-3^{+} (red bar) and DAPI^{+}/Caspase-3^{+} (grey bar) cells in *Cdkn1c*-MADM-7 (*MADM-7*$^{GT/TG,Cdkn1c}$;*Emx1*$^{Cre/+}$) with maternal deletion **(b)** and *Cdkn1c*-MADM-7 (*MADM-7*$^{GT/TG,Cdkn1c}$;*Emx1*$^{Cre/+}$) with paternal deletion **(d)**. Note that homozygote *Cdkn1c*$^{+/+}$ red cells show significantly increased survival when compared to heterozygous *Cdkn1c*$^{+/-}$ unlabeled DAPI^{+} cells, independent of UPD status. Bars indicate mean ±SEM. **p<0.01 (two-tailed T-test).

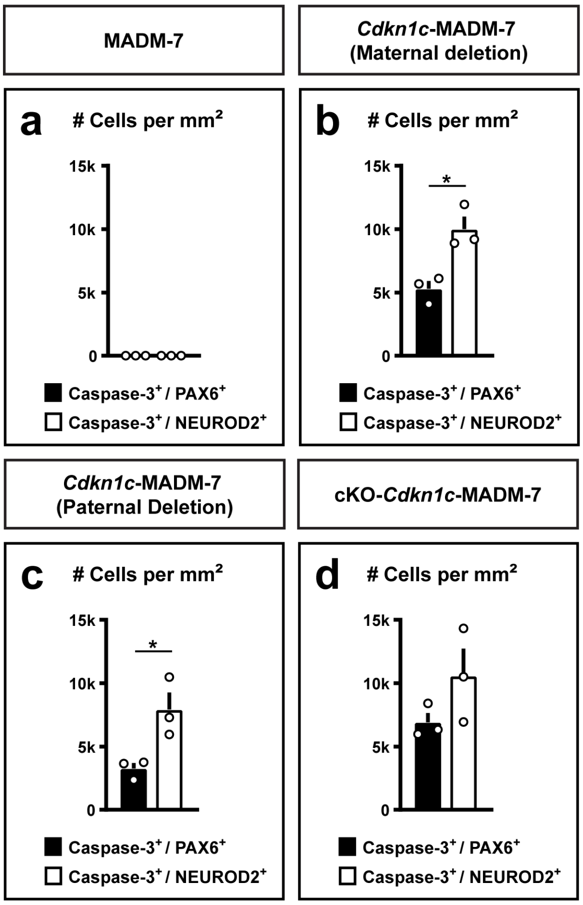

Supplementary Figure 8. Nascent projection neurons and RGPs require *Cdkn1c* for survival. (a-d) Analysis of Caspase-3^{+} figures in RGPs ($PAX6^{+}$) and projection neurons ($NEUROD2^{+}$) in MADM-7 (*MADM-*$7^{GT/TG}$*;Emx1*$^{Cre/+}$) **(a)**, *Cdkn1c*-MADM-7 (*MADM-*$7^{GT/TG,Cdkn1c}$*;Emx1*$^{Cre/+}$) with maternal deletion **(b)**, *Cdkn1c*-MADM-7 (*MADM-*$7^{GT/TG,Cdkn1c}$*;Emx1*$^{Cre/+}$) with paternal deletion **(c)**, and cKO-*Cdkn1c*-MADM-7 (*MADM-*$7^{GT,Cdkn1c/TG,Cdkn1c}$*;Emx1*$^{Cre/+}$) **(d)** in cerebral cortex at E13. Black bar represents number of Caspase-3^{+}/$PAX6^{+}$ cells per mm^2 (RGPs) and white bar Caspase-3^{+}/$NEUROD2^{+}$ cells per mm^2 (projection neurons) (k, thousand). Note that projection neurons show higher apoptosis rate when compared to RGPs. Data points indicate single individuals (n=3). Bars represent mean ±SEM. *p<0.05 (two-tailed T-test).

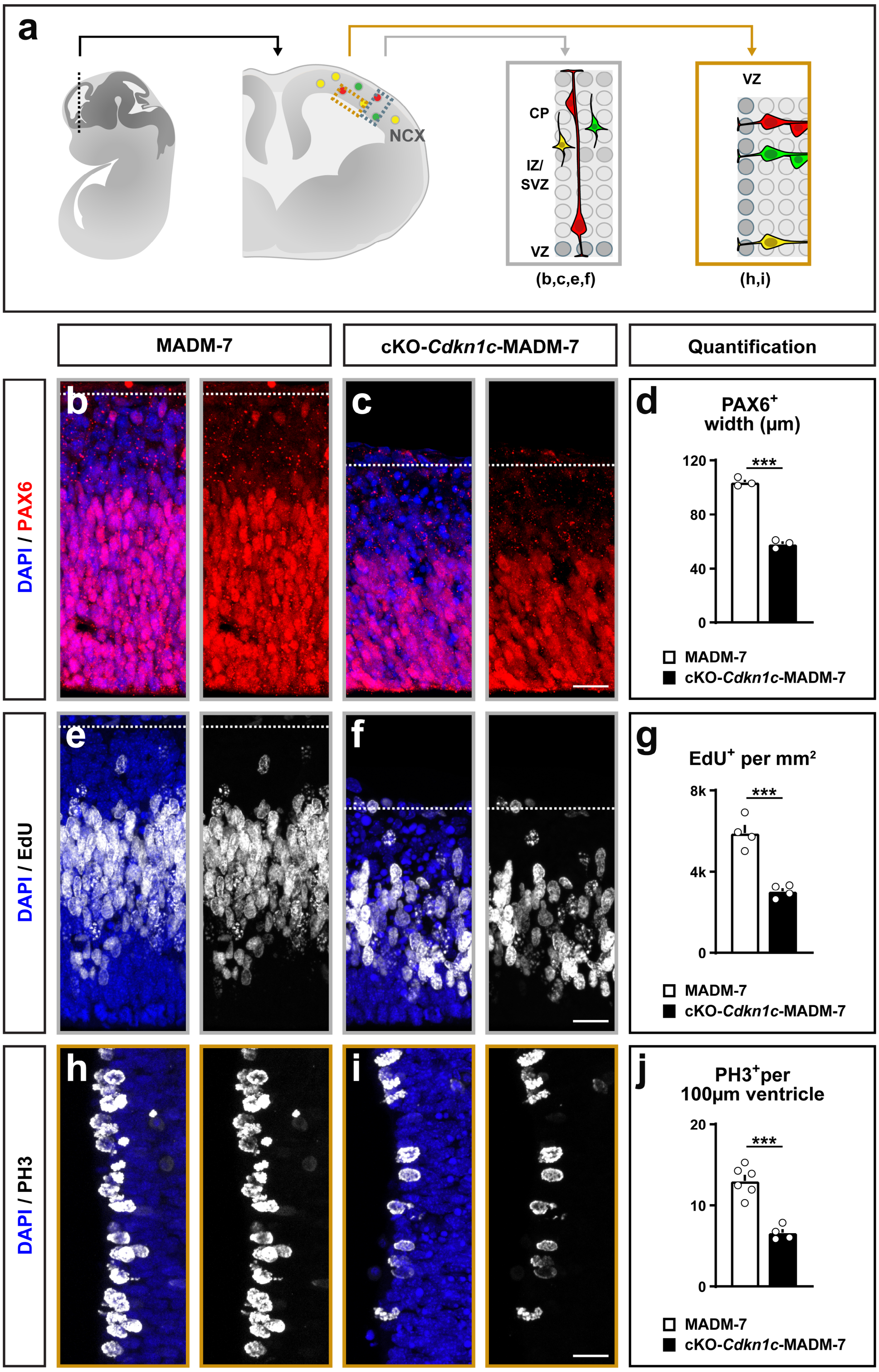

Supplementary Figure 9. Decreased population of proliferating RGPs in cKO-*Cdkn1c*. (a) Schematic depicts the analyzed areas in the developing E13 cortex. Scheme was created by the authors of this paper. **(b-j)** Analysis of PAX6 (red) **(b-d)** EdU (white) incorporation (1h chase) **(h-j)** and PH3 (white) **(e-g)** expression in MADM-7 (*MADM-7$^{GT/TG}$;Emx1$^{Cre/+}$*) **(b, e, h)** and cKO-*Cdkn1c*-MADM-7 (*MADM-7$^{GT,Cdkn1c/TG,Cdkn1c}$;Emx1$^{Cre/+}$*) **(c, f, i)**. Quantification **(d, g, j)** indicates average thickness (µm) of PAX6^{+} cell layer **(d)**, number of cells with incorporated EdU^{+} per mm^{2} **(g)**,and number of mitotic cells (PH3^{+}) per 100µm of ventricular wall **(j)**, in MADM-7 (white bar) and cKO-Cdkn1c-MADM-7 (black bar). Data points indicate single individuals **(d, j)** or individual hemispheres from a minimum of two individuals **(g)**. Bars indicate mean ±SEM. ***p<0.001 (two-tailed T-test). Nuclei were stained using DAPI (blue). Scale bar, 20 µm.

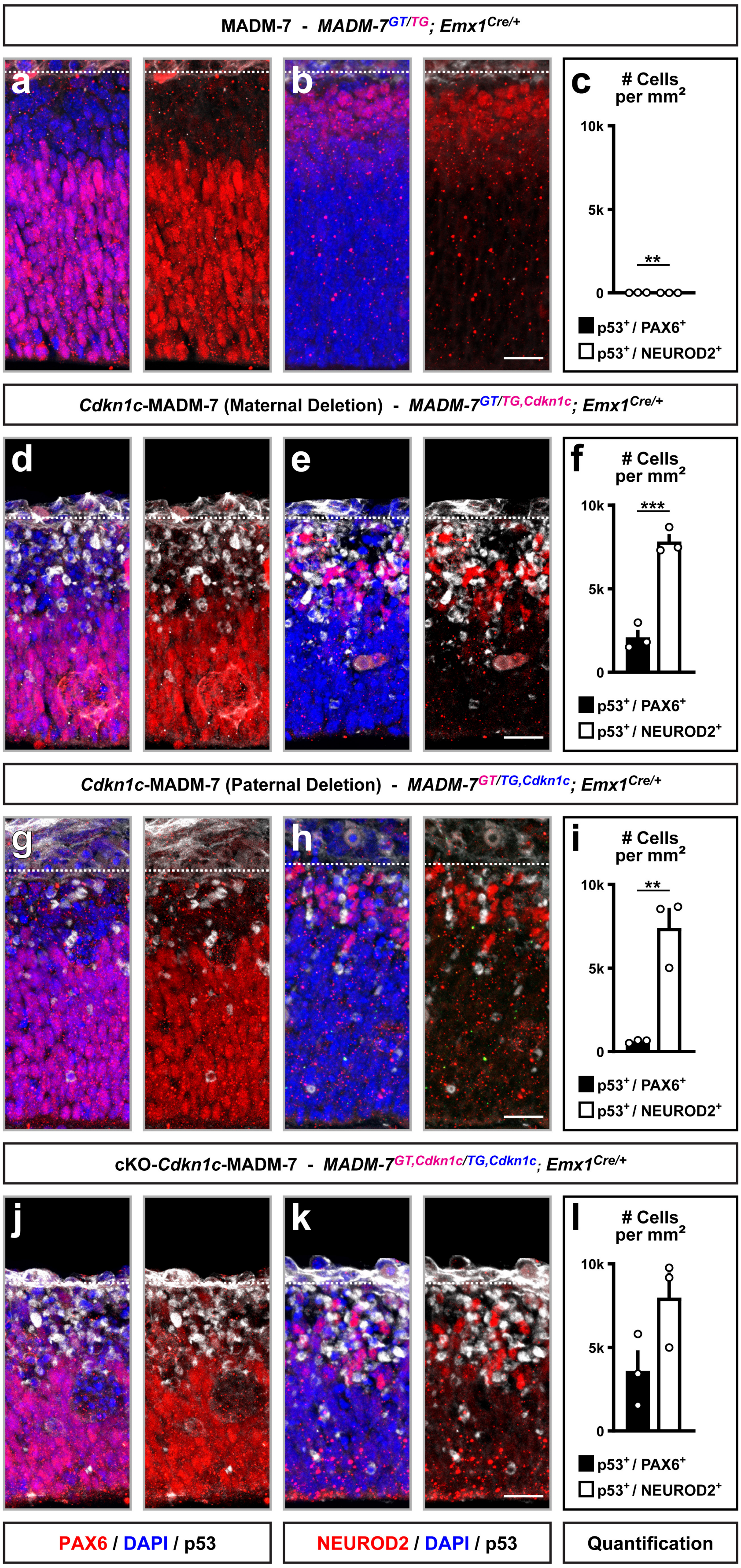
MADM-7 - MADM-7GT/TG; Emx1Cre/+
a
b
c
Cells per mm2
10k
5k
0
**
p53+ / PAX6+
p53+ / NEUROD2+
Cdkn1c-MADM-7 (Maternal Deletion) - MADM-7GT/TG,Cdkn1c; Emx1Cre/+
d
e
f
***
Cdkn1c-MADM-7 (Paternal Deletion) - MADM-7GT/TG,Cdkn1c; Emx1Cre/+
g
h
i
cKO-Cdkn1c-MADM-7 - MADM-7GT,Cdkn1c/TG,Cdkn1c; Emx1Cre/+
j
k
l
PAX6 / DAPI / p53
NEUROD2 / DAPI / p53
Quantification

Supplementary Figure 10. Upregulation of p53 upon genetic deletion of *Cdkn1c*. (a-l) Analysis of p53 expression (white) in RGPs (PAX6^{+}, red) **(a, d, g, j)** and projection neurons (NEUROD2^{+}, red) **(b, e, h, k)** in MADM-7 (*MADM-7$^{GT/TG}$;Emx1$^{Cre/+}$*) (**a-c**), *Cdkn1c*-MADM-7 (*MADM-7$^{GT/TG,Cdkn1c}$;Emx1$^{Cre/+}$*) with maternal deletion (**d-f**), *Cdkn1c*-MADM-7 (*MADM-7$^{GT/TG,Cdkn1c}$;Emx1$^{Cre/+}$*) with paternal deletion (**g-i**), and cKO-*Cdkn1c*-MADM-7 (*MADM-7$^{GT,Cdkn1c/TG,Cdkn1c}$;Emx1$^{Cre/+}$*) (**j-l**) at E13. The parent from which the MADM cassette, and with recombined *Cdkn1c-flox* allele (*Cdkn1c*) was inherited, is indicated in the respective genotypes in pink (mother) and blue (father). Quantification of p53^{+}/PAX6^{+} and p53^{+}/NEUROD2^{+} cells (k, thousand) per mm^{2} in MADM-7 **(c)**, *Cdkn1c*-MADM with maternal deletion **(f)**, *Cdkn1c*-MADM with paternal deletion **(i)**, and cKO-*Cdkn1c*-MADM **(l)**. Note that projection neurons (NEUROD2^{+}) have higher levels of p53 when compared to RGPs (PAX6^{+}). Data points indicate single individuals (n=3). Bars represent mean ±SEM. **p<0.01, ***p<0.001 (two-tailed T-test). Nuclei were stained using DAPI (blue). Scale bar, 20 µm.

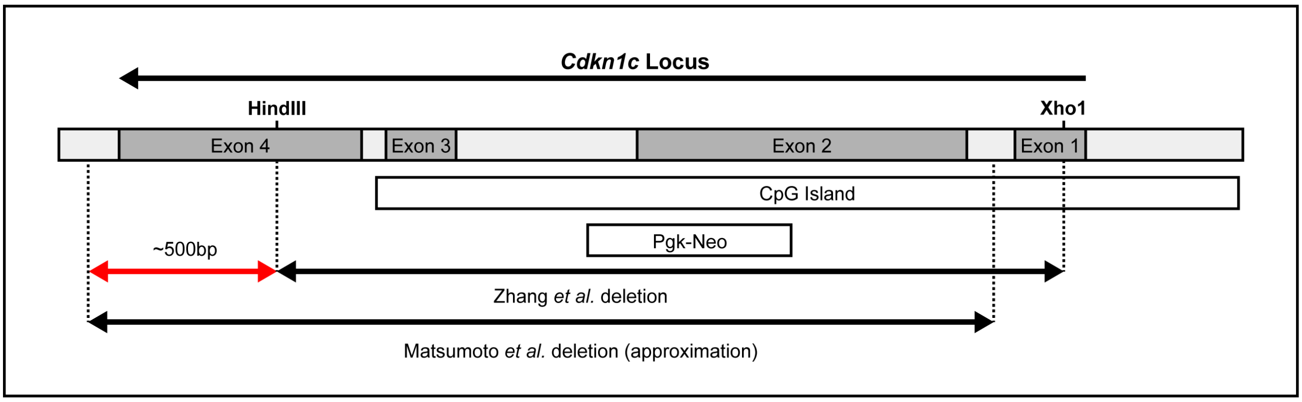

Supplementary Figure 11. *Cdkn1c* genomic locus architecture and structure of deletion alleles. Overview of genomic *Cdkn1c* locus with 4 exons. CpG island is indicated (white box). Two different *Cdkn1c* transgenic alleles[1,2] are depicted by black arrows. Restriction sites for HindIII and Xho1 are indicated and were used to create the deletion in the Zhang et al. allele [2]. Note that in the Zhang et al. allele [2] the Pgk-Neo cassette was not removed before analysis. Red arrow indicates an approximate 500bp region which is deleted upon conditional ablation of the Matsumoto et al. floxed-allele [1] but not in the Zhang et al. deleted allele [2].

Supplementary Table 1. Antibodies used in this study.

Primary antibodies:

**Name/Dilution**	**Company**	**Order number**
Chicken anti-GFP 1:500	Aves Labs	GFP-1020
Rabbit anti-RFP 1:500	MBL	PM005
Goat anti-tdTomato 1:500	Sicgen Antibodies	AB8181-200
Rabbit anti-Tbr1 1:500	Abcam	AB31940
Mouse anti-SatB2 1:100	Abcam	AB51502
Rabbit anti-Caspase-3 1:500	Cell Signaling	9662S
Rabbit anti-Caspase-3-A647 1:50	Cell Signaling	9602S
Rabbit anti-Ki67 1:200	Abcam	AB15580
Rabbit anti-NeuroD2 1:200	Abcam	AB109406
Rabbit anti-Pax6 1:500	Cell Signaling	60433S
Mouse anti-p53 1:100	Cell Signaling	48818
Rabbit anti-pH3 1:300	Cell Signaling	3377T

Secondary antibodies:

**Name/Dilution**	**Company**	**Order number**
Donkey Anti-Chicken Alexa Fluor 488 1:500	Jackson Immuno Research	715-475-150
Donkey Anti-Goat Cy3 1:500	Jackson Immuno Research	705-165-147
Donkey Anti-Rabbit Cy3 1:500	Jackson Immuno Research	711-165-152
Donkey Anti-Rabbit Alexa Fluor 647 1:500	Jackson Immuno Research	711-605-152
Donkey Anti-Mouse Alexa Fluor 647 1:500	Jackson Immuno Research	715-605-151
Donkey Anti-Rabbit DyLight 405 1:300	Jackson Immuno Research	711-475-152
Donkey Anti-Mouse DyLight 405 1:500	Jackson Immuno Research	715-475-150
Donkey Anti-Goat Alexa Fluor 568 1:100	Molecular Probes	A11057
Donkey Anti-Rabbit Alexa Fluor 647 1:1000	Molecular Probes	A31573
Donkey Anti-Rabbit Alexa Fluor 568 1:1000	Molecular Probes	A10042

Supplementary References

1 Matsumoto, A. *et al.* p57 is required for quiescence and maintenance of adult hematopoietic stem cells. *Cell stem cell* **9**, 262-271, doi:10.1016/j.stem.2011.06.014 (2011).

2 Zhang, P. *et al.* Altered cell differentiation and proliferation in mice lacking p57KIP2 indicates a role in Beckwith-Wiedemann syndrome. *Nature* **387**, 151-158, doi:10.1038/387151a0 (1997).
