# Supplementary material for: Imprinted Cdkn1c genomic locus cell-autonomously promotes cell survival in cerebral cortex development
Source: Nat Commun. 2020 Jan 10;11:195. doi: 10.1038/s41467-019-14077-2 (PMC6954230; doi:10.1038/s41467-019-14077-2)
Supplement: Supplementary file 8 — Reporting Summary [file 41467_2019_14077_MOESM8_ESM.pdf]

nature research

Corresponding author(s): Simon Hippenmeyer

Last updated by author(s): Dec 3, 2019

Reporting Summary

Nature Research wishes to improve the reproducibility of the work that we publish. This form provides structure for consistency and transparency in reporting. For further information on Nature Research policies, see Authors & Referees and the Editorial Policy Checklist .

Statistics

For all statistical analyses, confirm that the following items are present in the figure legend, table legend, main text, or Methods section.

n/a	Confirmed	
☐	☒	The exact sample size (*n*) for each experimental group/condition, given as a discrete number and unit of measurement
☐	☒	A statement on whether measurements were taken from distinct samples or whether the same sample was measured repeatedly
☐	☒	The statistical test(s) used AND whether they are one- or two-sided<br>*Only common tests should be described solely by name; describe more complex techniques in the Methods section.*
☒	☐	A description of all covariates tested
☒	☐	A description of any assumptions or corrections, such as tests of normality and adjustment for multiple comparisons
☐	☒	A full description of the statistical parameters including central tendency (e.g. means) or other basic estimates (e.g. regression coefficient) AND variation (e.g. standard deviation) or associated estimates of uncertainty (e.g. confidence intervals)
☒	☐	For null hypothesis testing, the test statistic (e.g. *F*, *t*, *r*) with confidence intervals, effect sizes, degrees of freedom and *P* value noted<br>*Give P values as exact values whenever suitable.*
☒	☐	For Bayesian analysis, information on the choice of priors and Markov chain Monte Carlo settings
☒	☐	For hierarchical and complex designs, identification of the appropriate level for tests and full reporting of outcomes
☒	☐	Estimates of effect sizes (e.g. Cohen's *d*, Pearson's *r*), indicating how they were calculated

Our web collection on statistics for biologists contains articles on many of the points above.

Software and code

Policy information about availability of computer code

Data collection	All software used for data collection is described in detail in the methods section.
Data analysis	All software and code used for data analysis is described in detail in the methods section.

For manuscripts utilizing custom algorithms or software that are central to the research but not yet described in published literature, software must be made available to editors/reviewers. We strongly encourage code deposition in a community repository (e.g. GitHub). See the Nature Research guidelines for submitting code & software for further information.

Data

Policy information about availability of data

All manuscripts must include a data availability statement. This statement should provide the following information, where applicable:

- Accession codes, unique identifiers, or web links for publicly available datasets
- A list of figures that have associated raw data
- A description of any restrictions on data availability

Deep sequencing data will be submitted to a public repository before publication. A supplementary table containing raw data is provided in the manuscript.

Field-specific reporting

Please select the one below that is the best fit for your research. If you are not sure, read the appropriate sections before making your selection.

☒ Life sciences ☐ Behavioural & social sciences ☐ Ecological, evolutionary & environmental sciences

For a reference copy of the document with all sections, see nature.com/documents/nr-reporting-summary-flat.pdf

Life sciences study design

All studies must disclose on these points even when the disclosure is negative.

Sample size	No statistical method was used to determine sample size. Variations between samples were used to determine appropriateness of sample size. In addition, sample sizes were based upon previously reported data.
Data exclusions	Nothing to declare
Replication	All replications were consistent.
Randomization	Groups were assigned based on genotype.
Blinding	For the quantification of MADM-labeled cells investigators were blinded to group allocation during data collection and analysis.

Reporting for specific materials, systems and methods

We require information from authors about some types of materials, experimental systems and methods used in many studies. Here, indicate whether each material, system or method listed is relevant to your study. If you are not sure if a list item applies to your research, read the appropriate section before selecting a response.

Materials & experimental systems

n/a	Involved in the study	
☐	☒	Antibodies
☒	☐	Eukaryotic cell lines
☒	☐	Palaeontology
☐	☒	Animals and other organisms
☒	☐	Human research participants
☒	☐	Clinical data

Methods

n/a	Involved in the study	
☒	☐	ChIP-seq
☐	☒	Flow cytometry
☒	☐	MRI-based neuroimaging

Antibodies

Antibodies used

See also methods section.

Chicken anti-GFP 1:500 Aves Labs GFP-1020
Rabbit anti-RFP 1:500 MBL PM005
Goat anti-tdTomato 1:500 Sicgen Antibodies AB8181-200
Rabbit anti-Tbr1 1:500 Abcam AB31940
Mouse anti-SatB2 1:100 Abcam AB51502
Rabbit anti-Caspase-3 1:300 Promega G7481
Rabbit anti-Caspase-3 1:500 Cell Signaling 9662S
Rabbit anti-Caspase-3-A647 1:50 Cell Signaling 9602S
Rabbit anti-Ki67 1:200 Abcam AB15580
Rabbit anti-NeuroD2 1:200 Abcam AB109406
Rabbit anti-Pax6 1:500 Cell Signaling 60433S
Mouse anti-P53 1:100 Cell Signaling 48818
Rabbit anti-pH3 1:300 Cell Signaling 3377T
Donkey Anti-Chicken Alexa Fluor 488 1:500 Jackson Immuno Research 715-475-150
Donkey Anti-Goat Cy3 1:500 Jackson Immuno Research 705-165-147
Donkey Anti-Rabbit Cy3 1:500 Jackson Immuno Research 711-165-152
Donkey Anti-Rabbit Alexa Fluor 647 1:500 Jackson Immuno Research 711-605-152
Donkey Anti-Mouse Alexa Fluor 647 1:500 Jackson Immuno Research 715-605-151
Donkey Anti-Rabbit DyLight 405 1:300 Jackson Immuno Research 711-475-152
Donkey Anti-Mouse DyLight 405 1:500 Jackson Immuno Research 715-475-150
Donkey Anti-Goat Alexa Fluor 568 1:100 Molecular Probes A11057
Donkey Anti-Rabbit Alexa Fluor 647 1:1000 Molecular Probes A31573
Donkey Anti-Rabbit Alexa Fluor 568 1:1000 Molecular Probes A10042

Validation

All validation is provided by the commercial vendors.

Animals and other organisms

Policy information about studies involving animals; ARRIVE guidelines recommended for reporting animal research

Laboratory animals	See also methods section.<br><br>Mouse lines:<br>Mouse: MADM-7-GT The Jackson Laboratory IMSR_JAX: 021457<br>Mouse: MADM-7-TG The Jackson Laboratory IMSR_JAX: 021458<br>Mouse: Emx1-Cre The Jackson Laboratory IMSR_JAX: 005628<br>Mouse: Cdkn1c-flox (Matsumoto et al. 2011)<br>Mouse: CAST/EiJ The Jackson Laboratory IMSR_JAX: 000928<br>Mouse: C57BL/6J The Jackson Laboratory IMSR_JAX: 000664
Wild animals	n/a
Field-collected samples	n/a
Ethics oversight	Mouse protocols were reviewed by institutional preclinical core facility (PCF) at IST Austria and an institutional ethics committee. All breeding and experimentation was performed under a license approved by the Austrian Federal Ministry of Science and Research in accordance with the Austrian and EU animal laws.

Note that full information on the approval of the study protocol must also be provided in the manuscript.

Flow Cytometry

Plots

Confirm that:

- [ ] The axis labels state the marker and fluorochrome used (e.g. CD4-FITC).
- [ ] The axis scales are clearly visible. Include numbers along axes only for bottom left plot of group (a 'group' is an analysis of identical markers).
- [ ] All plots are contour plots with outliers or pseudocolor plots.
- [ ] A numerical value for number of cells or percentage (with statistics) is provided.

Methodology

Sample preparation	See methods section.
Instrument	FACS Aria III BD Biosciences
Software	BD FACSDIVA Software or FlowJo Software
Cell population abundance	See methods section.
Gating strategy	See methods section.

- [ ] Tick this box to confirm that a figure exemplifying the gating strategy is provided in the Supplementary Information.
