# Supplementary material for: Imprinted Cdkn1c genomic locus cell-autonomously promotes cell survival in cerebral cortex development
Source: Nat Commun. 2020 Jan 10;11:195. doi: 10.1038/s41467-019-14077-2 (PMC6954230; doi:10.1038/s41467-019-14077-2)
Supplement: Supplementary file 9 — Description of Additional Supplementary Files [file 41467_2019_14077_MOESM9_ESM.pdf]

Title: Supplementary Data 1. Sample details for transcriptome analysis of MADM-labeled cells.
Description: This table lists relevant meta information for samples analyzed in Figure 1c and Figure 2b.

Title: Supplementary Data 2. Differential gene expression analysis of MADM-labeled cells.
Description: This table provides the output of the DESeq2 analysis of samples detailed in Supplementary Data 1 separately for E13 and E16 time points. Analysis details are provided in the Methods.

Title: Supplementary Data 3. Sample details for transcriptome analysis of *Cdkn1c* cKO.
Description: This table lists relevant meta information for samples analyzed in Figure 4.

Title: Supplementary Data 4. Differential gene expression analysis of *Cdkn1c* cKO.
Description: This table provides the output of the DESeq2 analysis of samples detailed in Supplementary Data 3. Analysis details are provided in the Methods.

Title: Supplementary Data 5. Gene ontology analysis of up-regulated (a) and down-regulated genes (b) in *Cdkn1c* cKO.
Description: This table shows all significantly enriched GO terms (details see Methods). For simplicity of display in Figure 4f the long list of enriched terms was reduced by REVIGO (eliminated = 1, see Methods for details) or manually (manually.eliminated = 1). Only terms with final = 0 are shown in Figure 4f.
